# Deep Learning Based Monitoring of Spatter Behavior by the Acoustic Signal in Selective Laser Melting

**DOI:** 10.3390/s21217179

**Published:** 2021-10-28

**Authors:** Shuyang Luo, Xiuquan Ma, Jie Xu, Menglei Li, Longchao Cao

**Affiliations:** 1 School of Mechanical Science and Engineering, Huazhong University of Science & Technology, Wuhan 430074, China; LuoSY@hust.edu.cn (S.L.); xma@hust.edu.cn (X.M.); 13125198826@163.com (J.X.); LML1058011747@163.com (M.L.); 2 School of Aerospace Engineering, Huazhong University of Science & Technology, Wuhan 430074, China

**Keywords:** selective laser melting process, acoustic signal, deep learning, Spatter, monitoring

## Abstract

As one of the most promising metal additive manufacturing (AM) technologies, the selective laser melting (SLM) process has high expectations ofr its use in aerospace, medical, and other fields. However, various defects such as spatter, crack, and porosity seriously hinder the applications of the SLM process. In situ monitoring is a vital technique to detect the defects in advance, which is expected to reduce the defects. This work proposed a method that combined acoustic signals with a deep learning algorithm to monitor the spatter behaviors. The acoustic signals were recorded by a microphone and the spatter information was collected by a coaxial high-speed camera simultaneously. The signals were divided into two types according to the number and intensity of spatter during the SLM process with different combinations of processing parameters. Deep learning models, one-dimensional Convolutional Neural Network (1D-CNN), two-dimensional Convolutional Neural Network (2D-CNN), Recurrent Neural Network (RNN), Long Short Term Memory (LSTM), and Gated Recurrent Unit (GRU) were trained to establish the relationships between the acoustic signals and characteristics of spatter. After K-fold verification, the highest classification confidence of models is 85.08%. This work demonstrates that it is feasible to use acoustic signals in monitoring the spatter defect during the SLM process. It is possible to use cheap and simple microphones instead of expensive and complicated high-speed cameras for monitoring spatter behaviors.

## 1. Introduction

The selective laser melting (SLM) process is considered to be one of the most promising additive manufacturing (AM) technologies due to its unique manufacturing capabilities, such as fast forming speed, freedom of design, and its ability to manufacture complex components [1]. SLM is a metal-based powder bed AM technology including the manufacturing process of design, printing, post-processing, and evaluation. There are high expectations for its use in many fields such as medical, aerospace, and automobile industries.

Although the SLM process is the most promising technology of AM, the generation of defects during the SLM process is the biggest limitation for its wide application. These defects include the spatter, balling, porosity, and so on [2,3]. As one of the most common defects, spatter directly affects the interaction between the laser and material that may cause a failure of the building process. Spatter that falls on the surface of the powder bed or the solidified layer will form cohesive particles, thus affecting the quality of the following layer and result in internal defects. Spatter can be divided into droplet spatter and powder spatter [4]. The droplet spatter is caused by the instability of the molten pool surface. The powder spatter results from the blowing of unmelted powder around the molten pool [5]. The form factors of these two types of spatter can be attributed to vaporization and the recoil pressure on the surface of the molten pool [6].

In recent years, various sensors and methods have been built to monitor the spatter. Khairallah et al. [7] established a 3D high-precision powder scale numerical model. The generation mechanism of spatter-induced defects during SLM process was studied. They derived criteria to stabilize the melt pool dynamics and minimize defects, which provided great help to improve the reliability of SLM process. Tan et al. [8] used a high-speed camera to capture spatter. A segmentation method was proposed to distinguish spatter from the molten pool. Zhang et al. [9] monitored the SLM process using an off-axis high-speed camera. Kalman filter tracking technology was utilized to locate the molten pool accurately. An image segmentation algorithm was proposed to differentiate molten pool, plume, and spatter. The relationship between spatter characteristics and scanning strategy was studied. Gunenthiram et al. [6] used a high-speed camera to study the formability of 316L stainless steel powder and A4047 aluminum powder. They found that the spatter increased with the increase of volume energy density. For low volume energy density values, spatter was incorporated into the molten pool whereas above a volume energy density threshold generating more or less intense vaporization, a possible key-hole regime and accelerated liquid flow backwards, such spatter was accelerated sheared upwards by the metallic gas plume and ejected. They found 316L stainless steel powder had more spatter than A4047 aluminum powder under the same process conditions. Zhang et al. [10] studied a digital stripe projection technology, which can be used to detect the location and size of spatter during SLM process.

Restricted by the limitations of clamping position, angle, and distance, a single high-speed camera cannot monitor comprehensive aspects of the SLM process [11]. Except for optical signals, the SLM process is also accompanied by acoustic signals and thermal signals [12,13]. Microphone sensors have been applied to monitoring acoustic signals due to the advantages of lower cost, non-contact, non-destructive, and flexible [14,15]. Shevchik et al. [16] used an fiber Bragg grating sensor to record the acoustic signals during SLM process. An optical microscopy-based approach was proposed to define different types of porosity. A deep learning algorithm based model was proposed to monitor the SLM process by acoustic signals. Ye et al. [17] used two dimension-reduction algorithms to extract features of acoustic signals. The Support Vector Machine (SVM) was utilized to identify the defects. A method based on a deep belief network (DBN) was proposed to study the relationship between acoustic signals and five melting states [5]. Cheng et al. [18] used a microphone to record acoustic signals of laser-assisted ceramic additive manufacturing. The acoustic signals were utilized to monitor the part forming process by combining a data processing algorithm.

With the rapid development of machine learning algorithms, more and more intelligent algorithms were applied to the process monitoring of AM [19]. Zhang et al. [20] developed an image processing tool to automatically extract porosity information from signals collected by a high-speed camera. A Convolutional Neural Network (CNN) model was built to predict the porosity. Scime et al. [21] used a high-speed camera to capture the molten pool images. An unsupervised learning algorithm was proposed to distinguish the molten pool and identify defects. Okaro et al. [22] used photodiodes to collect data and a semi-supervised machine learning algorithm was proposed to automatically identify defects during the SLM process. The results showed that the proposed algorithm could still get accepted results with reduced data samples. Shevchik et al. [23] used a fiber Bragg grating sensor to detect the airborne acoustic emission (AE) signals. The spectral convolutional neural network (SCNN) was applied to differentiate the acoustic characteristics of dissimilar quality (poor, medium, high part quality defined according to porosity). Wasmer et al. [24] proposed a quality monitoring method that combined reinforcement learning algorithm with acoustic signals. Gobert et al. [25] developed an in-situ defect monitoring strategy for SLM process. They adopted a high-resolution digital single-lens reflex camera to collect images. The SVM was applied to extract features and classify defects. The accuracy of the proposed monitoring strategy is 85%. Coeck et al. [26] used an off-axis melt pool monitoring system to obtain molten pool data. The correlation between the molten pool and defects was established to predict the location of lack of fusion porosities.

In this work, a low-cost and high sampling rate microphone is used to monitoring the SLM process. The relationship between acoustic signals and spatter captured by a high-speed camera is built. A deep learning algorithm is proposed to monitor the spatter with a low-cost microphone instead of a high-speed camera. This paper makes three main contributions: (a) An image segmentation method based on contour extraction algorithm was proposed to distinguish molten pool and spatter. (b) The characteristics of acoustic signals during SLM process was analyzed based on short-time Fourier transform (STFT) and fast Fourier transform (FFT). (c) The correlations between acoustic signals and spatter were established base on deep learning algorithm. The feasibility of using acoustic signals for defect monitoring in SLM process is demonstrated.

The rest of this paper is organized as follows. In Section 2, an experimental device is designed to verify the proposed monitoring method. In Section 3, the method of signal pre-processing and a neural network are establised. In Section 4, the experimental results are discussed. In addition, the performances of various neural network models are compared, followed by the conclusion and future work in Section 5.

## 2. Experimental Setup and Datasets

### 2.1. Experimental Setup and Material

In this study, 316L stainless steel powder was utilized for experiments. This material is widely used in 3D printing due to its advantages of good weldability [27]. The particle sizes of the powder are mainly between 15~37 μm. Its chemical composition is shown in Table 1.

The experiments were carried out using a metal 3D printer (FF-M140). The printing machine is equipped with a continuous fiber laser (C300L) with a beam quality factor M2 < 1.3. The wavelength of the laser is 1064 nm and the range of the spot diameter is from 50~80 μm. The maximum output power is 250 W and the maximum scanning speed can reach 7000 mm/s. The main parameters of the SLM equipment are listed in Table 2.

The image of spatter during the SLM process are captured by a coaxial high-speed camera (IDT NX4-S3) made by IDT from Pasadena, CA, USA. The maximum frame rate is up to 22,500 fps with a corresponding resolution of 128 pixel × 128 pixel. The maximum resolution can reach 1024 pixel × 1024 pixel when the frame rate is 3000 fps. The coaxial high-speed camera is mounted above the build chamber through a custom window. To obtain enough information per second while ensuring a high image resolution, a compromise should be reached between the frame rate and the resolution of the high-speed camera. The frame rate of the high-speed camera is set at 10,000 fps with a resolution of 256 pixel × 256 pixel by trial and error. The high-speed camera is controlled by motion studio software during the SLM process.

**Figure 1:**
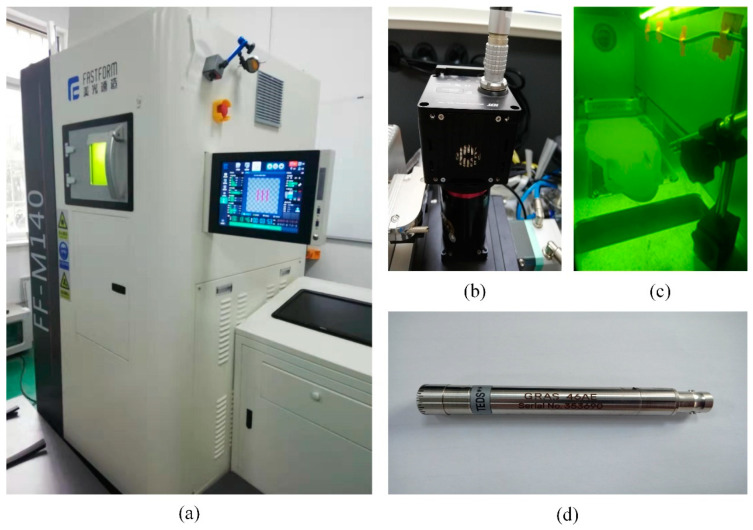
The setup and arrangement of the experiments: (**a**) The SLM equipment, (**b**) the high-speed camera, (**c**) the arrangement of the microphone in the build chamber, and (**d**) the microphone sensor.

**Figure 2:**
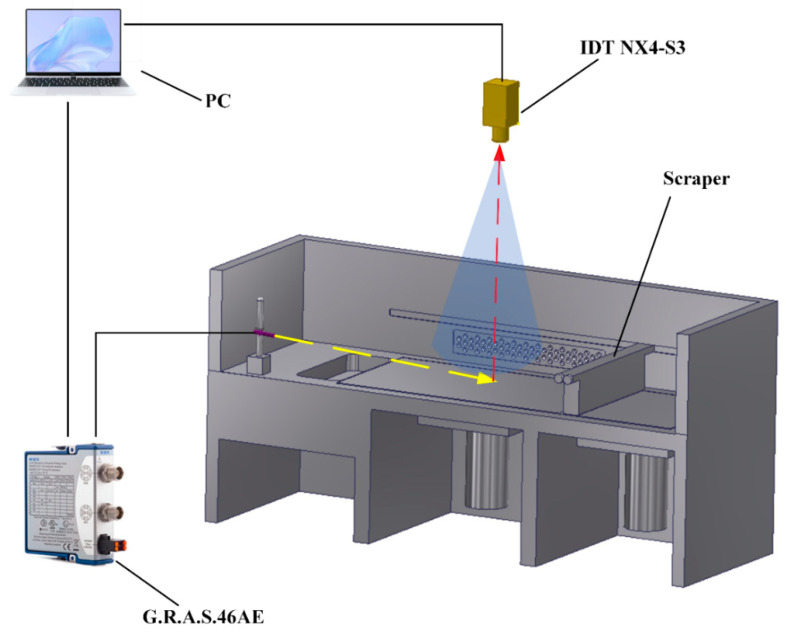
Schematic of microphone sensor and coaxial high speed camera monitoring system for the SLM process.

In this study, the G.R.A.S. 46AE 1/2″ CCP free-field standard microphone was utilized to acquire the acoustic signals during the SLM process. This type of microphone can work in a harsh environment. It has a high response frequency of 3.15~51.2 kHz. The performance is related to the angle and relative position of the microphone. As shown in Figure 1c, a microphone clamping device was designed. This device can adjust the angle and relative position to ensure the microphone to collect enough acoustic signals. In this work, the microphone was installed 20 cm away from the building substrate with an angle of 45° facing the center of the processing substrate. The microphone was connected via a NI-9218 data acquisition card. Labview software was used to set up the acquisition parameters. The Schematic of microphone sensor and coaxial high speed camera monitoring system for the SLM process is shown in Figure 2.

### 2.2. Data Acquisition

**Figure 3:**
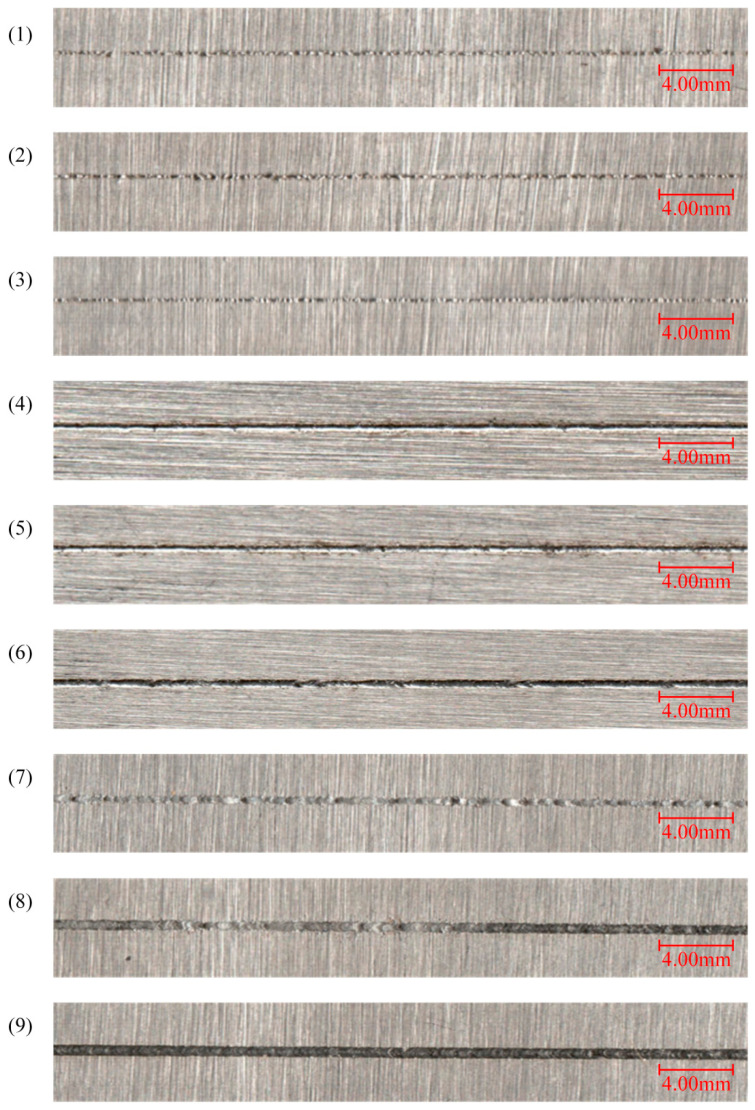
The morphology of the single tracks of 9 groups process parameters.

To investigate the relationship between the acoustic signals and spatter, acoustic signals and spatter images under different process conditions are collected. Table 3 shows the combinations of different process parameters. Nine groups of single tracks experiments were conducted. The laser power changed from 25 W to 225 W with an interval of 25 W. The scanning speed was kept at 30 mm/s. The thickness of the powder bed was kept the same. The length of each track was set at 60 mm and the distance between the two adjacent tracks was 20 mm. Figure 3 demonstrates the morphology of the single tracks of nine groups process parameters.

## 3. Methodology

### 3.1. The Acoustic Signals during SLM Process

During the SLM process, the spatter is mainly caused by recoil pressure and surface tension [28]. The acoustic signal is caused by pressure waves during the metal vapor and plasma plume ejecting from the molten pool. The dynamic variation of the plasma plume leads to the variation of recoil pressure and surface tension. Plasma density *N_p_* is determined as:

(1) $$N_{P}=3N_{M}d\Delta N\exp(-(E_{n}-\varphi )/kT)$$

where *d* is the diameter of focused laser radiation, Δ is the cross-section of the atom, $E_{n}$ represents atomic ionization potential, φ is the work function of the metal. The expression for k is kd=π/2. The plasma density $N_{p}$ goes up as ion density N increases. Vapor density $N_{M}$ increases as surface temperature T increases. The plasma density $N_{P}$ affects the atmospheric pressure $P_{0}$, thus affecting the acoustic signal [5]. The acoustic intensity *I*_n_ is expressed as:

(2) $$I_{n}=\frac{P_{0}^{2}}{f(N_{p})v_{e}}$$

where the air density f() is the function of plasma density, and $v_{e}$ represents the acoustic signal propagation speed. The intensity of the acoustic signal collected by the microphone near the molten pool is related to the plasma plume. Therefore, the acoustic signal is related to spatter during the SLM process from the theoretical derivation.

### 3.2. In-Situ Data Processing

#### 3.2.1. Preprocessing of Spatter Image

**Figure 4:**
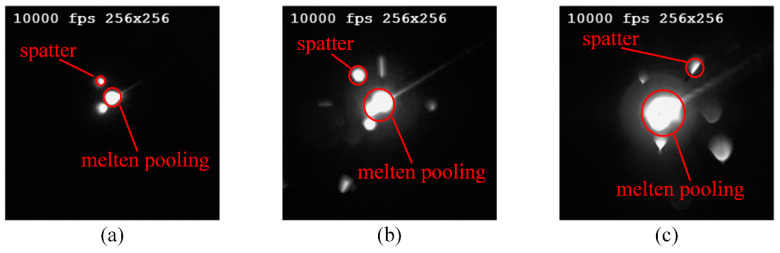
Typical images obtained by coaxial high-speed camera with different laser powers: (**a**) 25 W, (**b**) 125 W, and (**c**) 225 W.

To extract feature information from the spatter images, an image processing method is needed to extract spatter features. In this work, an image processing method based on contour extraction and area comparison was proposed to process the spatter images. Two kinds of features, the number and the intensity of spatters, were extracted. As shown in Figure 4, the brightness value of the molten pool and spatter are very similar while the area of the molten pool is significantly larger than that of the spatter. Thus, the molten pool can be removed form a image by comparing their areas to avoid the influence of molten pool.

**Figure 5:**
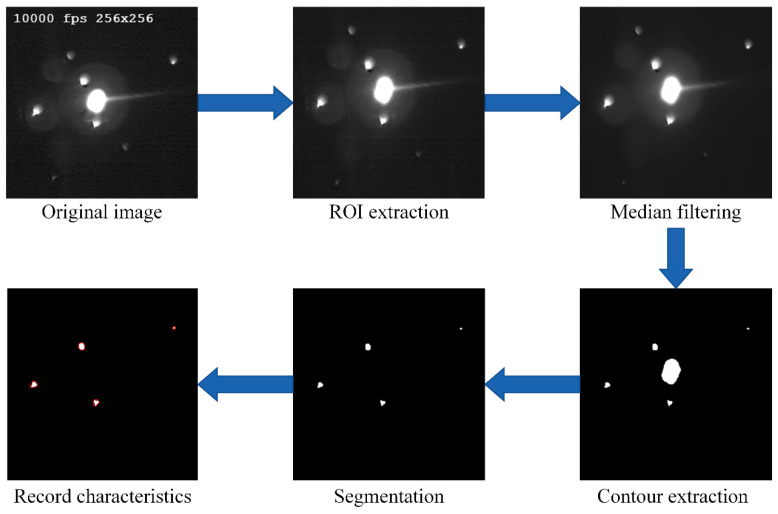
Steps of feature extraction for the spatter. (original image—ROI extraction—median filtering—record characteristics—segmentation—contour extraction).

The steps of feature extraction of the spatters are shown in Figure 5. Firstly, the region of interest (ROI) is extracted to get rid of irrelevant factors from an original image. Meanwhile, the sizes of the images are reduced by cutting the irrelated parts to improve the processing efficiency. Secondly, a median filter method is used to remove the noise. All the contours are determined through the connected domain using the contour extraction algorithm in Opencv to obtain the area of each contour. Thirdly, a feature map of a spatter is extracted by removing the contour with the maximum area after comparing the area of each contour. Finally, the number and intensity of the spatters are calculated.

Binarization processing is used to extract the contour of the spatter. It is important to determine the threshold value of binarization. If the threshold is set too high, the sensitivity of weak spatter will be decreased. On the contrary, part of the spatter will be overlapped, increasing the recording error of the number of spatters. In this work, the threshold of binarization value is set as 240 to reduce the error. For feature determination, the number and intensity of spatters (the number of pixels per spatter) are extracted. Due to the laser power will change the number and intensity of spatters, the weighted fusion of the two factors as the label is used to increase the accuracy.

#### 3.2.2. Preprocessing of Acoustic Signals

**Figure 6:**
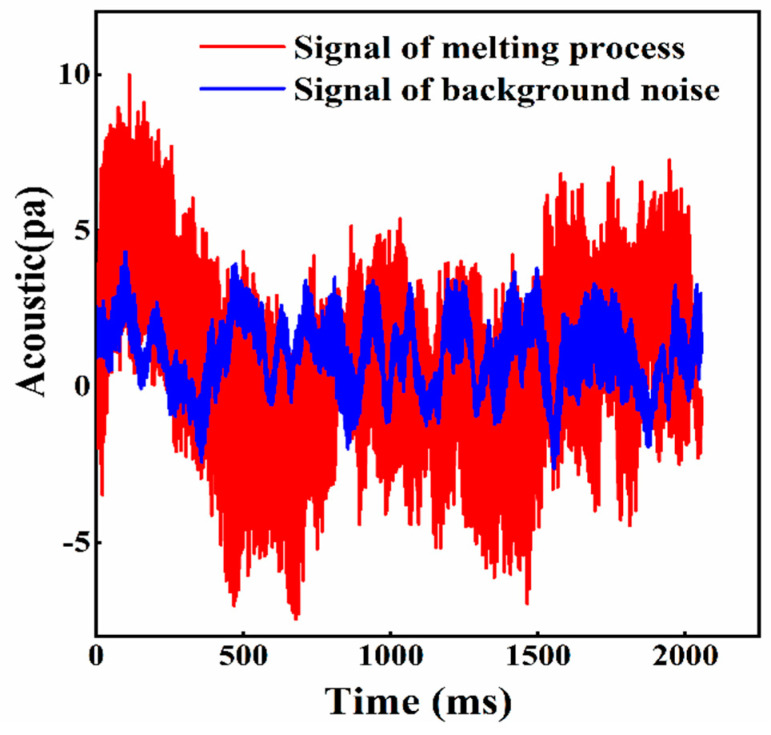
Acoustic signals collected of ambient noise (laser shutdown) and melting process (scanning speed 30 mm/s and laser power 200 W).

Figure 6 shows the time-domain diagram of the acoustic signals of the melting process of powder (red line) and before melting (blue line) with the same laser power of 200 W. The amplitude of the acoustic signal during the melting process is higher and the waveform is obviously different. Hence, the melting process can be easily distinguished from the time domain diagram.

SLM process includes powder feeding, powder spreading, powder melting, and solidifying. To extract the acoustic signal during the SLM, the short-time Fourier transform (STFT) is used to analyze the signal in the time-frequency domain. The formula of short-time Fourier transform is expressed as:

(3) $$X(n,\omega )=\sum_{m=-\infty }^{\infty }x(m)\omega (n-m)e^{-j\omega (m)}$$

where x(m) represents the input signal and ω(m) is the window function. x(n,ω) is a two-dimensional function of time n and frequency ω [18]. The Hann window function is applied to slid along the time axis. It performs Fourier transformation on the collected signals and restores them to the time domain, thus obtaining the time-frequency diagram of the signal. The length of the window function was set as 256 and the overlapping area was half of the length.

**Figure 7:**
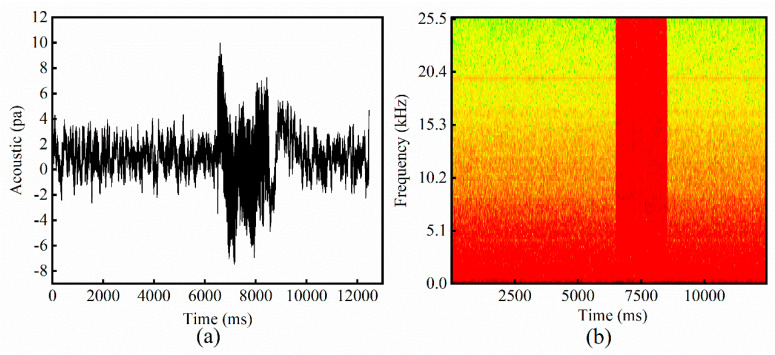
The STFT of sound signal during SLM: (**a**) Time-domain diagram of the sound signal (The red box illustrates the window function), and (**b**) the time-frequency diagram after STFT transformation. (scanning speed 30 mm/s and laser power 200 W).

The time-frequency diagram after STFT transformation is shown in Figure 7. The amplitude of the high-frequency component is small before the laser is turned on. When the powder is being melted, a large amount of high-frequency characteristic is generated, so the starting and ending points of the processing can be determined. It indicates that the amplitude of the low-frequency component is always large because of background noise. A high-pass filter is used to process the signal, and the filtering value is set at 2 kHz to reduce the interference of background noise.

#### 3.2.3. Convolutional Neural Network

In this section, a brief introduction of CNN model is presented. Deep learning is a complex machine learning algorithm that aims to make machines as analytical as humans and able to recognize data such as text, images, and sound [29,30]. As a classic network of deep learning, CNN has a specific structure. It mainly consists of input layer, convolutional layer, pooling layer, and output layer. One-dimensional CNN (1D CNN) is suitable for extracting the features of sequence data like acoustic signal.

**Figure 8:**
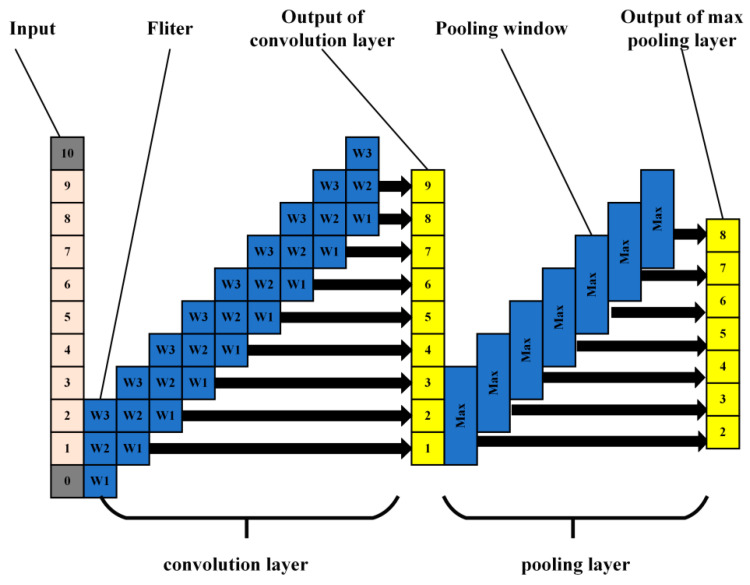
The schematic diagram of the one-dimensional CNN. (fliter denotes feature extractor, max denotes max pooling and wi denotes weigh matrix).

Figure 8 shows the schematic diagram of the one-dimensional CNN. The fliter denotes feature extractor, max denotes max pooling, and wi denotes weigh matrix. The acquired one-dimensional signals of specific length were used as inputs. The one dimensional input signals are scanned by feature extractor to obtain the discrete convolution results. The convolution kernel is a weight matrix. Pooling mainly includes max pooling and average pooling. Max pooling extracts the maximum value of each pooling window, and average pooling extracts the average value. In this paper, the most commonly used max pooling was used to scan the input discrete convolution results. Then a new feature map was obtained.

The main function of the convolutional layer is to reduce the dimension and extract features of input data by a convolution operation. The inputs of the CNN model are a long vector, and the vector value is the amplitude of the acoustic signal after noise reduction. A convolutional layer usually contains several characteristic planes. Each characteristic plane is composed of some neurons. The neurons in the same characteristic plane share the same weights, which is called convolution kernel. The convolution kernel is usually initialized in the form of a random decimal matrix. In the training process, the convolution kernel will be trained to obtain reasonable weights.

The essence of weight-sharing is feature extraction, as well as reducing the risk of overfitting. The convolution formula is as follows [20,31,32]:

(4) $$y^{conv}=f((W*X)+b)$$

where $y^{conv}$ represents the output value matrix of the convolutional layer and f() represents the activation function. Common activation functions include Sigmoid function, Tanh function, ReLU function, etc. W is the convolution kernel weight coefficient matrix, X is the input matrix, and b is the bias coefficient matrix.

The reduction of dimension and feature extraction of input data is accomplished by a convolution operation. To further improve the computing speed and robustness of feature extraction, a value to replace the characteristics of a region is used in the pooling process. Pooling methods are generally divided into maximum pooling and average pooling. The formula of max-pooling is:

(5) $$y^{pool}=\max(w(s_{1},s_{2})\cap y^{conv})$$

where $y^{pool}$ represents the output matrix of the pooling layer, $w(s_{1},s_{2})$ is the pooling window. $s_{1}$ and $s_{2}$ are the length and width values of the pooling window respectively.

Generally, the CNN model is composed of multiple convolutional layers and pooling layers. After a series of convolutional operations, the final feature map is inputted into the full connection layer, which acts as a classifier in the whole convolutional neural network. The formula of the full connection layer is:

(6) $$y=f(W_{f}y_{f}+b_{f})$$

where y is the classification result of the full connection layer, f() is the activation function, $W_{f}$ is the weight coefficient matrix, $y_{f}$ is the feature extraction result, and $b_{f}$ represents the bias coefficient matrix of the full connection layer.

## 4. Results and Discussion

### 4.1. Analysis of Spatter Image

**Figure 9:**
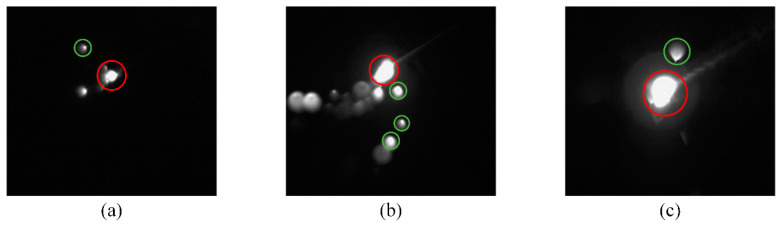
The collected typical spatter images with diffirent laser powers: (**a**) 50 W, (**b**) 100 W, and (**c**) 225 W. Molten pool is marked by the red circle while spatter is marked by green circles.

A high-speed camera was used to capture images of the SLM process. The method proposed in Section 2 was used to segment the molten pool and spatters. Figure 9 shows the spatters at a certain moment of different laser power (50 W, 100 W, and 225 W). It demonstrates that the area of the molten pool increases with the growth of laser power. While the number of spatters increases firstly and then decreases with the growth of the laser power. The spatter behavior can be divided into three categories with the increase of laser power. The number and intensity of spatters are lessened (as shown in Figure 9a) when the laser power is too low to completely melt the powder. When the laser power reaches a threshold, the number of spatters greatly increased (as shown in Figure 9b). As the laser power exceed the threshold, the spatters reduces (as shown in Figure 9c). The reason is the keyhole is not formed and the molten pool is too shallow to trapped the massively increased spatters. As the laser power continues to increase, the molten pool is penetrated deep enough. As a result, part of the spatter is restrained. This phenomenon is consistent with the results of the work of Gunenthiram et al. [6].

**Figure 10:**
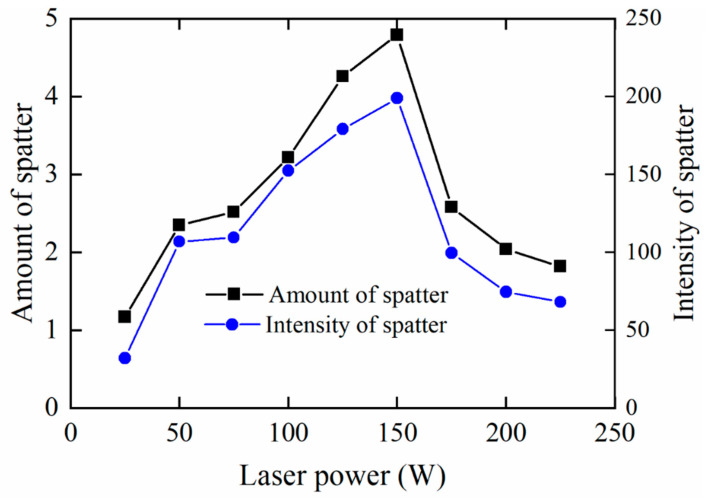
The number and intensity of spatter change with laser power during the SLM (the scaning speed is 30 mm/s).

Figure 10 shows the change in the number and intensity of spatters with the laser power. The number of spatters is the average value of the number of spatters from 100 successive images, and the spatter intensity is also the average value. It indicates that the number and intensity of spatters increase with the increase of laser power when it is less than 150 W. When the laser power is higher than 150 W, the number and intensity of the spatter decrease with the increase of the laser power. The number and intensity of spatters have the same trend as the laser power changes.

In this work, the scanning speed is fixed at 30 mm/s, while laser power is set to 25 W to 225 W with a interval of 25 W. Therefore, different energy density can be obtained with different laser powers. The energy density is defined as:

$$E=\frac{P}{\pi r^{2}\cdot v}$$

where *P* is the laser power, *r* is the radius of the focusing spot, and *v* is the scanning speed. Numerous studies indicate that there is close relationships between the spatter and the energy density during the SLM process [1,2,3,4,5]. It implies that when the scanning speed and the focusing spot are constant, the laser power has great influence on the spatter. The spatter can be classified into two types according the generation mechanism: droplet spatter caused by the recoil pressure and surface tension and powder spatter caused by blast wave at the front of the molten pool [1]. A low laser power means a low energy density which is not capable of melting sufficient power as shown in Figure 3a–c. So the number and intensity of spatters are low. As the laser power grows, the melted powder and molten pool increase. Thus, the increased recoil pressure causes more spatters. When the laser power reaches 150 W, the metal powder is over-melting with the growing energy density. Therefore, the number and intensity of spatters increase with the growth of the laser power. However, as the laser power increases from 150 W to 225 W, the number and intensity of spatters reduce gradually. There are three reasons for this behavior: (a) The droplet spatter fails to tear away from the molten pool at a high level of energy density [3]. (b) The substrate is melted as laser power reaches a high level. Thus, the viscosity of the molten pool is increased, which reduces the spatter. (c) When the energy density exceed a threshold, a relatively sable keyhole is built.

**Figure 11:**
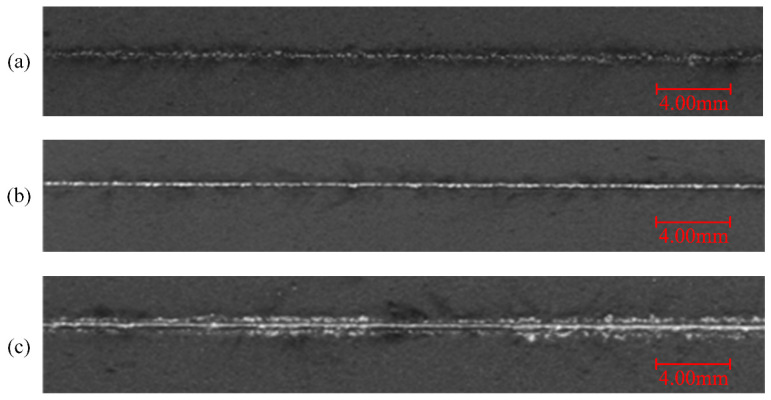
Morphology comparison of single tracks with different laser powers: (**a**) 25 W, (**b**) 100 W, and (**c**) 175 W. (the scanning speed is 30 mm/s).

As can be seen in Figure 11, when the laser power is 25 W, the spatter has little influence on the surrounding powder. The recoil pressure is not enough when the energy density is low. When the laser power increased to 100 W, the spattering areas become larger and denser, and the powder quality besides the weld track will be affected. As the laser power continues to increase, the range of spattering increases, but the density of spatters decreases, which is in line with the curve of the spatter with laser power.

### 4.2. Analysis of Acoustic Signal

**Figure 12:**
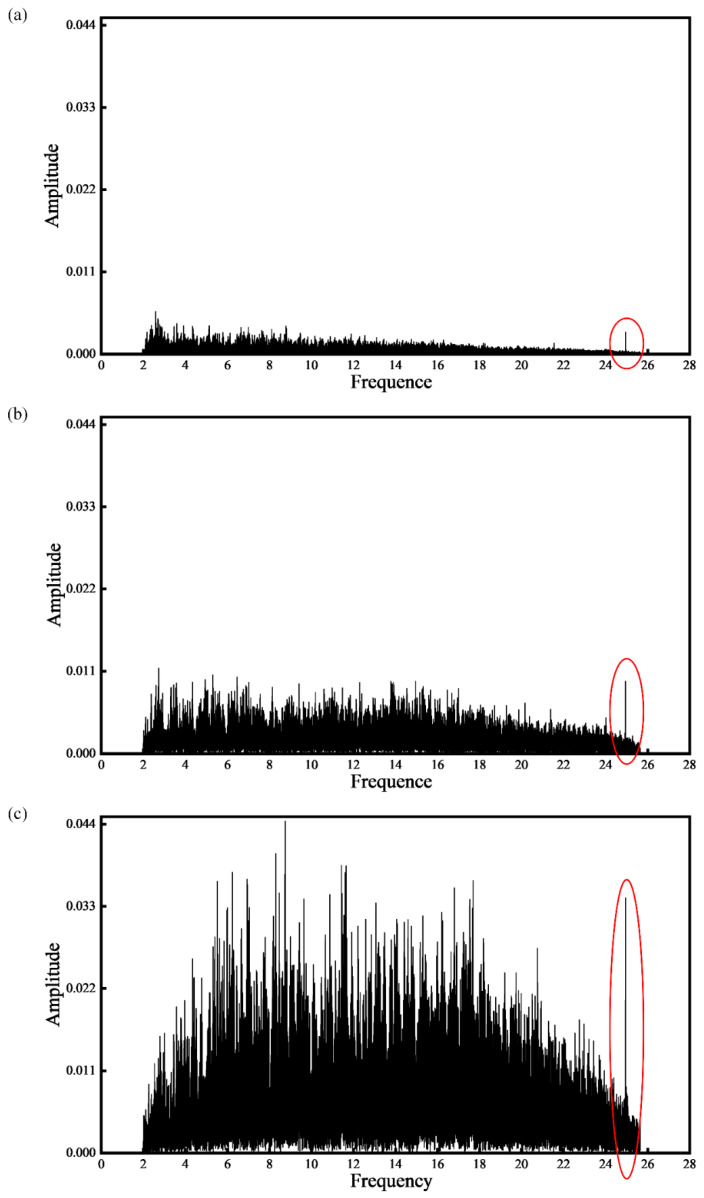
The frequency domain diagrams of sound signals with different laser powers: (**a**) 75 W; (**b**) 125 W and (**c**) 225 W. (the scaning speed is 30 mm/s).

The collected acoustic signals are processed using the proposed method in Section 2. Firstly, the acoustic signals during the SLM process are separated. Then the time-domain acoustic signals are converted into frequency-domain with fourier transform. Figure 12 shows the frequency domain diagrams of sound signals with different laser powers of 75 W, 125 W, and 225 W, respectively. The frequency of 0–2 kHz is filtered by a high-pass filter. With the increase of laser power, the amplitude of each frequency band also increases. As the maximum sampling rate of the microphone sensor is 51.2 kHz, the maximum frequency that can be analyzed is 25.6 kHz. There is an peak value at the position about 25 kHz, as marked by red circles in Figure 12. This frequency value can be considered to be closely related to the SLM process.

### 4.3. Classification of the Spatters

#### 4.3.1. Data Partitioning

**Figure 13:**
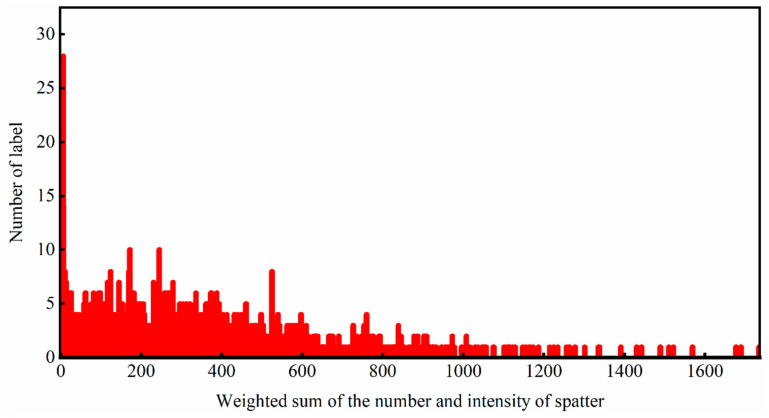
The histogram of the weighted sum of the number and intensity of spatter during SLM process.

The acoustic signal has a close relationship with the spatter during the SLM process. Acoustic signals are generated by the pressure wave when the droplet or plasma is ejected from the molten pool by recoil pressure. In this work, the frame rate of the high-speed camera and the sampling rate of microphone sensor are set to 10,000 fps and 51.2 kHz, respectively. To obtain enough training samples and sufficient information in each training sample, a compromise value was taken for the length of each sample. To avoid overlapping area, 512 original acoustic signal data points after denoising were taken as training sets successively with a step size of 512. Meanwhile, 100 frames of spatter images at the same time are utilized as the training labels. A sudden change in the intensity of spatters indicates a defect at that moment. The location of the defect can be determined based on the scanning speed and processing time. The datasets were divided into two parts (“high” and “low”) according to the median value of the weighted sum of the number and intensity of spatters. Figure 13 The histogram of the weighted sum of the number and intensity of spatters during the SLM process. It indicates that a large number of samples exist in the part with a low weighted sum value. The separation of the dataset is based on the intermediate value of the weighted sum of spatters. The number of total samples is 1809.

#### 4.3.2. Construction of the Proposed CNN

**Figure 14:**
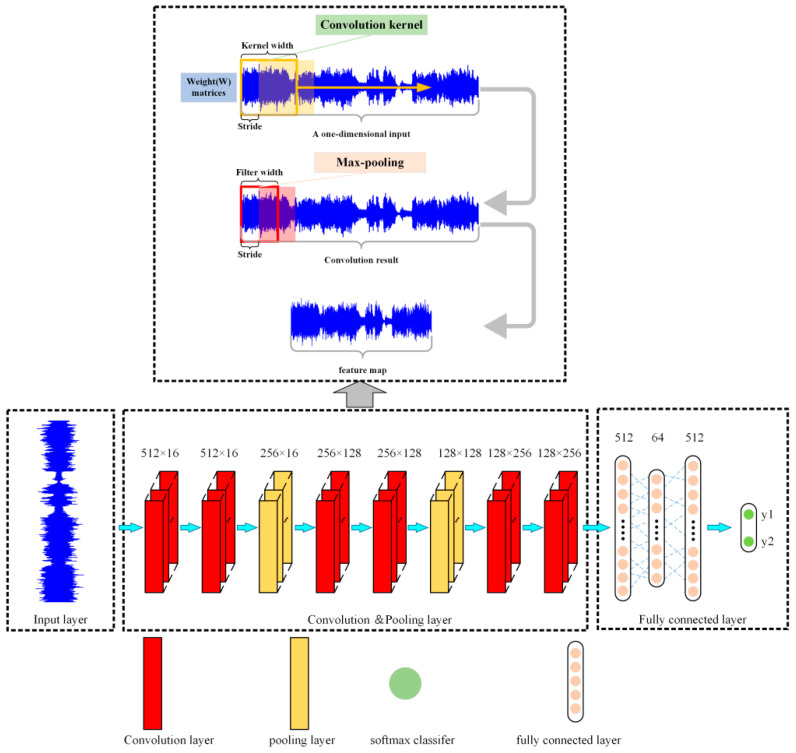
The structures of the proposed 1D CNN model.

To build the relationships between the acoustic signals and the spatter images during SLM process. A deep learning algorithm is used to classify and predict the spatters. A one-dimensional convolutional neural network is built. Figure 14 shows the structure of the proposed model. It is composed of three main parts: Input layer, convolution and pooling layer, and full connection layer. The convolution & pooling layer consists of six convolution layers and two pooling layers.

The one-dimension acoustic signals were the inputs while the spatter images are the outputs. As shown in Figure 14, each kernel includes 512 data points from the one-dimensional sample (acoustic signal) with a stride of 256 data points. Then, the features are extracted in the first and second convolution layers. The length and depth of the convolution kernel are both 512 and 16. After the convolutional process, feature data are input into the pooling layer to reduce dimensionality. The subsequent convolution and pooling operations are used for deep feature extraction. A Flattening operation is performed after feature extraction. Then all the feature maps are inputted into the fully connected layers. Finally, the classification results are outputted by the softmax classifier. Adam optimizer is adopted in the whole process. The learning rate with a initial value of 1 × 10^−4^ is set as self-adaptive decline to find the global optimal solution quickly. The number of epochs is set as 500 and the early-stopping mechanism is adopted. The binary-cross entropy function is selected as the loss function.

#### 4.3.3. Results of Classification

**Figure 15:**
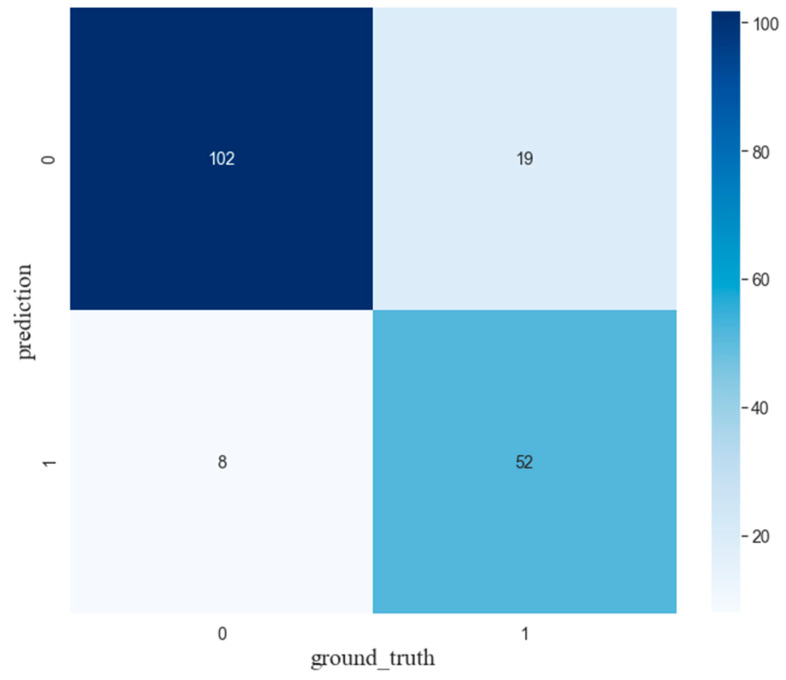
The confusion matrix of the test data set of original acoustic signal classification.

To train the proposed model, 90% of the data is randomly selected, and the remaining 10% is utilized to test and validate. K-fold cross-validation was selected for error estimation, and the classification accuracy was 85.08%. The confusion matrix of the test data is shown in Figure 15. Due to background noise caused by water-cooling machine, the powder feeder system, and the ventilation device, etc., high-frequency noise still exists. The accuracy of classification can reach 85.08 which is enough to reveal their relevance. The imbalance of two types of the datasets is also one of the major factors that affect the classification accuracy. The confusion matrix indicates that misclassification with high intensity of spatter accounts for a large proportion and it has a great influence on the accuracy. This problem can be optimized in future work.

**Figure 16:**
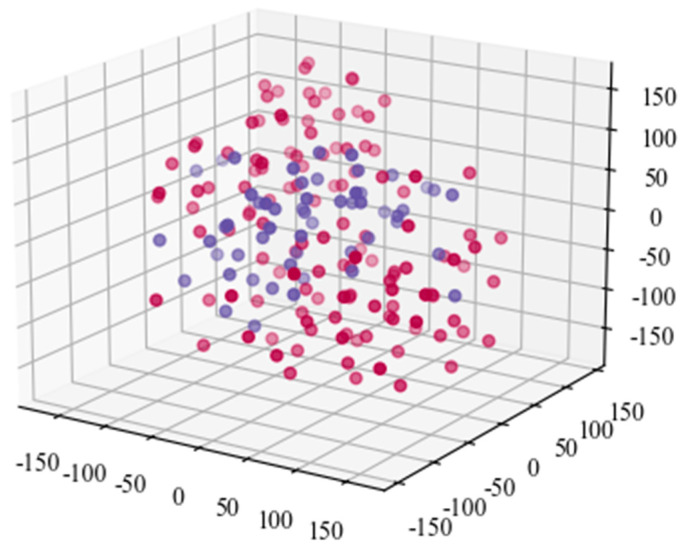
The diagram of the collected acoustic signal data after TSNE processing.

**Figure 17:**
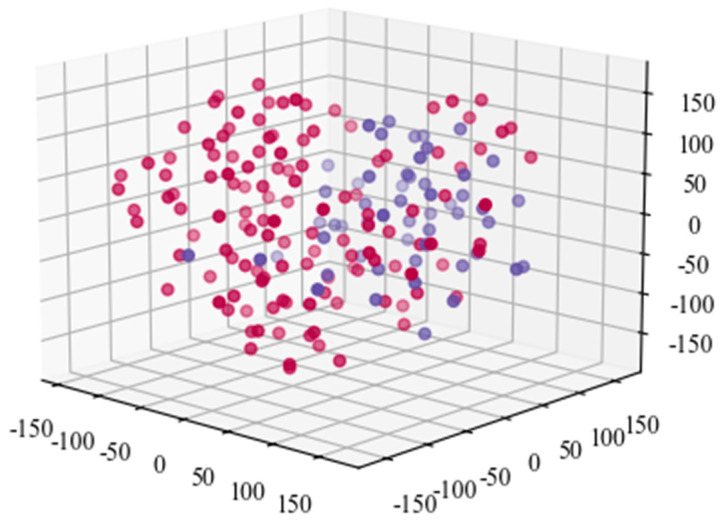
The diagram of the extracted acoustic signal characteristic data after TSNE processing.

To to verify and analyze the effectiveness of the proposed model, the TSNE (T-Stochastic Neighbor Embedding) is applied. Figure 16 shows the diagram of original acoustic signal data after TSNE processing. It demonstrates that the TSNE has poor capacity for classification based on the original acoustic signal data. To improve the accuracy, the extracted feature data by the convolutional layer of the proposed model is taken as the input of TSNE. Figure 17 is the extracted feature data processed by the TSNE model. It can be seen that there is an obvious boundary between the two types of datasets. It implies that the trained model can effectively extract and distinguish the characteristics of the signals.

To further explore the applicability of the model and the best classification method, the original, FFT, and two-dimensional image preprocessing of acoustic signals were conducted, respectively. Meanwhile, three recurrent neural networks, namely RNN, LSTM, and GRU were applied to compare with the propsoed 1D CNN. RNN is very effective for sequential data. It can extract temporal and semantic information in data. Unlike traditional RNNs, an LSTM network is well-suited to learn from experience to classify, process and predict time series when there are very long time lags of unknown size between important events [20]. GRU can effectively solve the problem of gradient explosion and has a good effect on long sequence input [33].

Table 4 shows the average value of the classification results of each network. For 1D CNN, the classification accuracy using the FFT data is 81.78, which is 3.3% lower than using the raw acoustic signal. The reason may be that the signal length after FFT processing is reduced to half of the original signal length. As a result, the function of the model cannot be fully displayed. The effect of the recurrent neural network on the original acoustic signal is not ideal. On the contrary, when FFT data is used as input, the classification accuracy is relatively high. This indicates that for recurrent neural networks, the FFT data can establish a better correlation with the spatter. 1D CNN has the best classification effect with a simple model and short training time.

## 5. Conclusions and Future Work

This work explored the relationships between acoustic signals and spatters during SLM process. The G.R.A.S. microphone sensor is used to acquire acoustic signals, and the FFT method is used to analyze the acoustic signal. A coaxial high-speed camera is applied to capture the spatter behaviors. An image segmentation algorithm was proposed to extract the spatter features. A deep learning model was developed to classify the spatters.

With the growth of laser power, the amplitude of acoustic signals increase. A large number of high-frequency signals are also generated in the SLM process. The frequency around 25 kHz is closely related to the SLM process. It is suggested that this frequency point is related to the molten pool behavior. With the increase of laser power, the intensity and number of the spatters increase firstly and then decrease. The spatter can be suppressed when a high laser energy density is used to ensure that the melt pool is deep enough. The classification accuracy of 1D CNN model for high and low spatter intensity can reach 85.08%, which was significantly higher than RNN, LSTM, and GRU models. CNN is significantly better than the RNN inefficiency. The classification accuracy of high intensity of spatter is low, which is mainly caused by the unbalanced data and the poor generalization ability of the model for high intensity of spatters.

In the future, improvements will be made in two areas: sensor and signal processing. In terms of the microphone sensor, a higher response frequency is in urgent need. Furthermore, the other sensors and multisensor fusion methods should be proposed. Given the materials and parameters selected in this work, the different parameters (scan speed and layer thickness) or the high spattering rate of transition metals or superalloys (Ni and Cu specially) will be studied in future work.

## Figures and Tables

**Table 1:** Chemical composition of 316L stainless steel powder.

C	Si	Mn	S	P	Cr	Ni	Mo	Fe
0.03	1.00	2.00	0.01	0.02	17.5~18	12.5~13	2.25~2.5	Bal.

**Table 2:** Basic parameters of the SLM system.

Parameters	Value
Maximum print size	120 mm × 120 mm × 120 mm
Power range	25 W~250 W
Diameter of laser spot	50~80 μm
Protective gas	Argon
Maximum scanning speed	7000 mm/s

**Table 3:** Parameters of laser power and scanning speed.

No.	Laser Power (W)	Scanning Speed (mm/s)
1	25	30
2	50	30
3	75	30
4	100	30
5	125	30
6	150	30
7	175	30
8	200	30
9	225	30

**Table 4:** Comparison of different machine learning model.

Network Structure	Data Processing	Classification Rate (%)
1D CNN	Raw data	85.08
	Data after FTT	81.78
RNN	Raw data	75.69
	Data after FTT	81.22
LSTM	Raw data	77.90
	Data after FTT	83.98
GRU	Raw data	81.22
	Data after FTT	80.11
2D CNN	Data after transform	80.56

## Data Availability

In order to reach the presented data in the paper, one can contact with the author.

## Nomenclature

SLM	Selective Laser Melting
AM	Additive manufacturing
CNN	Convolutional Neural Network
RNN	Recurrent Neural Network
LSTM	Long Short Term Memory
GRU	Gated Recurrent Unit
SVM	Support Vector Machine
DBN	Deep Belief Network
STFT	Short-Time Fourier Transform
FFT	Fast Fourier Transform
ROI	Region of Interest
1D CNN	One-dimensional Convolutional Neural Network
2D CNN	Two-dimensional Convolutional Neural Network
TSNE	T-Distributed Stochastic Neighbor Embedding
